# A Functional Larynx Dissection Utilizing Mandibular Rotation: A Technical Report

**DOI:** 10.7759/cureus.80767

**Published:** 2025-03-18

**Authors:** Mario Loomis, Brandon Trevino, Bradley Engel, Kyle Stitle, Hayden Fanguy, Yashna Thakker, Nicholas Fong, Matt Mackler, Natalie Mendoza

**Affiliations:** 1 Department of Clinical Anatomy, Sam Houston State University College of Osteopathic Medicine, Conroe, USA; 2 Department of Clinical Medicine, Sam Houston State University College of Osteopathic Medicine, Conroe, USA

**Keywords:** functional larynx dissection, larynx dissection, larynx structure function, lateral pharyngeal larynx exposure, teaching larynx anatomy

## Abstract

In gross anatomy labs, the larynx is usually visualized with either a sagittal hemisection or a posterior disarticulation approach. Both fail to maintain the functional in-situ relationships between the intact larynx and adjacent structures. This makes it challenging for students to understand how suprahyoid and infrahyoid muscles, pharyngeal muscles, the tongue, epiglottis, and intrinsic laryngeal muscles are all involved with airway protection and swallowing. We describe a novel approach to cadaveric larynx exposure using a pharyngotomy and mandibular rotation technique, which keeps the laryngeal relationships to the laryngopharynx, oropharynx, tongue, and soft palate intact.

## Introduction

Teaching laryngeal anatomy in a way that ties structure to function is challenging. This is generally done with anatomical models or dissections removing the larynx entirely from the body, and then demonstrating the motion of its components [1]. This separates the larynx from its key interrelationships, however, which are part and parcel of its function. When laryngeal function is taught completely in situ, it is primarily for airway management skills training, and not for detailed anatomical teaching [2]. Such skills training also requires the use of fresh or Thiel-embalmed cadavers, an embalming technique that utilizes a more complex set of chemicals and steps to maintain more pliable tissues [3,4]. In most medical schools, where fully embalmed cadavers are used to teach medical students, the hemisection and disarticulation techniques are the primary approaches to the larynx, both of which fail to maintain an appreciation of structural relationships regarding airway protection and swallowing. A lateral pharyngotomy, used by surgeons for the removal of oropharyngeal tumors, was the basis of our novel mandibular rotation exposure of the larynx [5]. In the cadaver, we extended the typical surgical incision through nerves and vessels that would not normally be transected in surgery, adding mandibular osteotomies to allow for a 90-degree rotation of the anterior mandible, providing a complete visualization of the larynx while maintaining structural and functional relationships.

## Technical report

**Figure 1:**
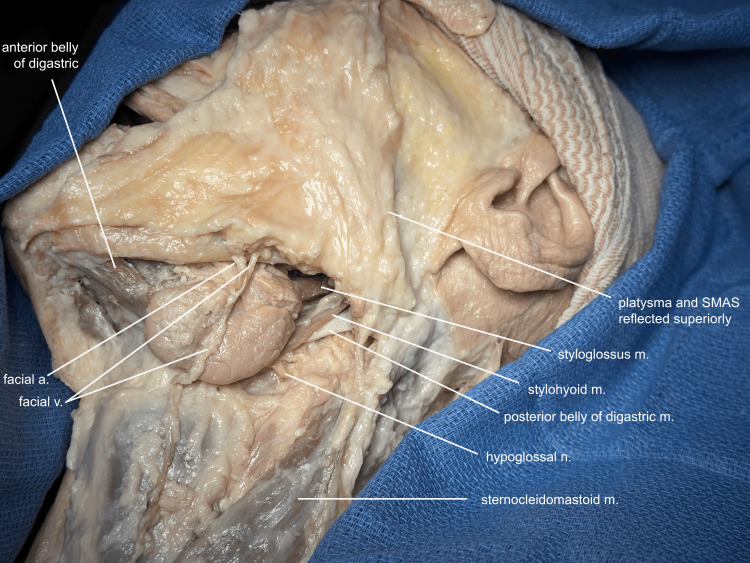
Submandibular dissection The platysma is elevated in continuity with the superficial aponeurotic system (SMAS) of the cheek, remaining below the tail of the parotid and facial nerve branches. The cervical fascia is opened to mobilize the submandibular gland and identify associated structures. a, artery; v, vein; m, muscle; n, nerve

After elevating a continuous skin flap over the cheek and neck, the platysma is elevated, beginning at the clavicle and extending superiorly over the mandibular border in continuity with the superficial musculoaponeurotic system (SMAS) of the lower cheek. The dissection plane remains deep to the branches of the facial nerve and, at the angle of the mandible, continues deep to the tail of the parotid gland. The superficial layer of the deep cervical fascia is then opened to mobilize the submandibular gland, taking care to identify the facial artery and vein that traverse it. The anterior and posterior bellies of the digastric, the stylohyoid, and the styloglossus muscles are likewise exposed and identified (Figure 1).

**Figure 2:**
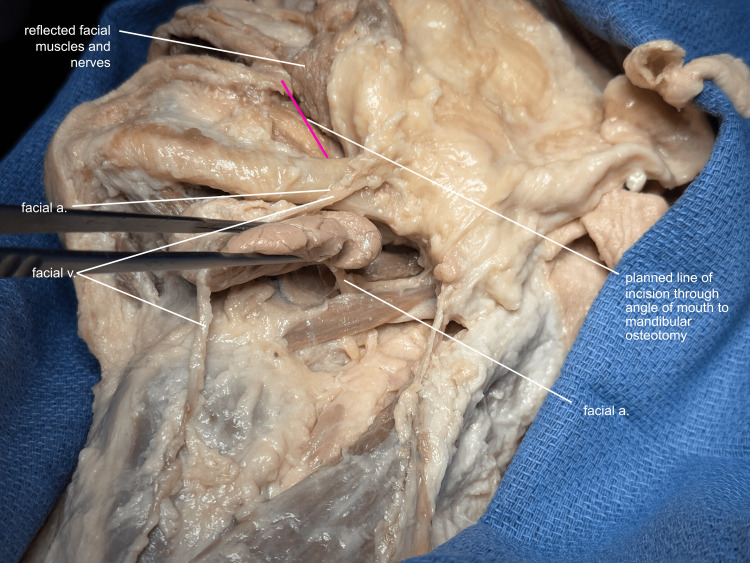
Preparing for intraoral incision Facial muscles and nerves are reflected to prepare for the incision from the angle of the mouth to the site of the mandibular osteotomy. a, artery; v, vein

The facial muscles and vessels are reflected superiorly and laterally, to expose the line of incision from the mandible to the corner of the mouth (Figure 2).

**Figure 3:**
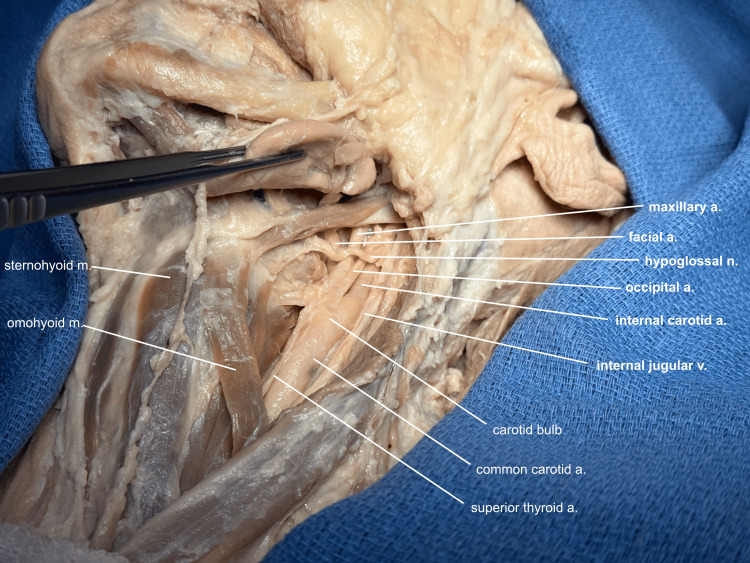
Additional neck dissection The cervical fascia is cleared further to reveal the strap muscles, vessels, and nerves of the neck. Note: This donor's occipital artery originated off the base of the external carotid, near the bulb. a, artery; v, vein; m, muscle; n, nerve

The submandibular gland is further mobilized, preserving the visceral efferent fibers and submandibular duct superiorly, and additional cervical fascia is cleared to reveal the strap muscles, neck vessels, and the hypoglossal nerve (Figure 3). 

**Figure 4:**
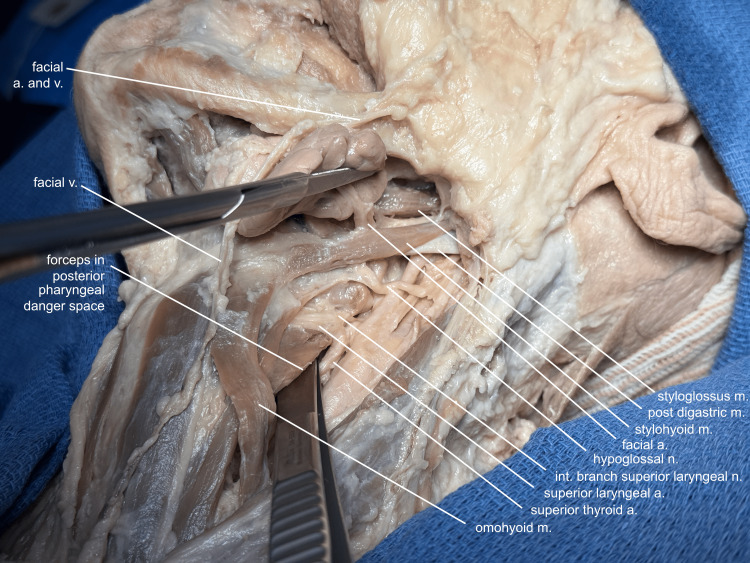
Structures to be divided Posterior to the laryngopharynx, the "danger space," so named because infections can spread through this space down to the mediastinum, is entered, and all the soft tissue structures needing to be divided to allow for mandibular rotation are thoroughly cleaned so that their identity can still be appreciated when the mandible is rotated back to its native position: the facial artery and vein, proximal and distal to the submandibular gland; the omohyoid muscle; superior thyroid artery; superior laryngeal artery; internal branch of the superior laryngeal nerve; hypoglossal nerve; and the stylohyoid, posterior digastric, and styloglossus muscles. Note: Usually, the lingual artery would also need to be divided, but in this donor, it came off the facial artery distal to the submandibular gland. Also, this donor's facial vein drained into the anterior, instead of the internal jugular. a, artery; v, vein; m, muscle; n, nerve

The loose areolar tissue posterior to the pharynx, termed the "danger space," is entered between the lateral edge of the thyrohyoid muscle and the superior thyroid artery by spreading the tissue posterior to the laryngopharynx. Structures needing to be divided for the larynx to rotate are cleaned and appreciated prior to transection, so they can be reapproximated and recognized when the mandible is rotated back to its native position (Figure 4). 

**Figure 5:**
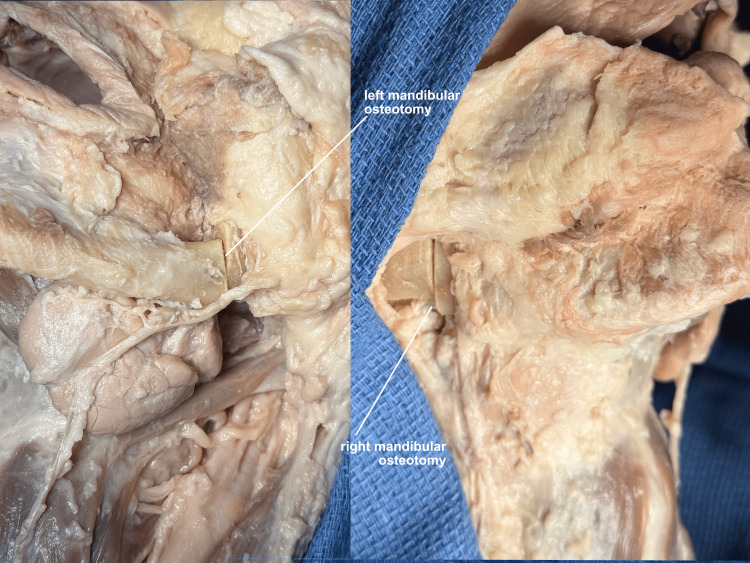
Mandibular osteotomies Symmetrical mandibular osteotomies are performed after elevating all soft tissue away from the bone, in the subperiosteal plane.

The body of the mandible is prepared for an osteotomy by elevating all soft tissue away from the bone just anterior to the masseter muscle, and an osteotomy is performed. The same is done on the opposite side (Figure 5).

**Figure 6:**
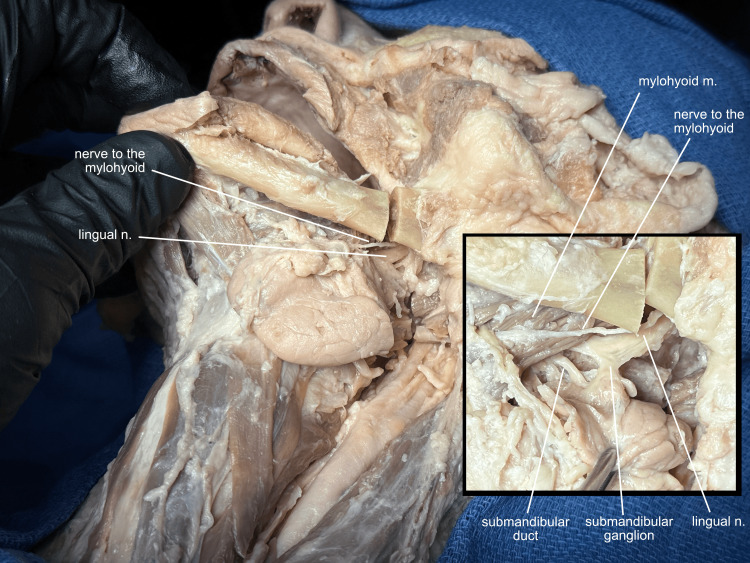
Mobilizing the osteotomy After dividing the neck structures, oral mucosa, and buccinator, the mandibular osteotomy is gently mobilized to allow for a clear view of the lingual nerve, nerve to the mylohyoid, submandibular ganglion, and submandibular duct. m, muscle; n, nerve

An incision is made from the angle of the mouth to the site of the mandibular osteotomy through the oral mucosa and buccinator muscle, and the neck structures are divided (omohyoid, superior thyroid artery 2 cm above the thyroid, internal branch of the superior laryngeal nerve and superior laryngeal artery, hypoglossal nerve, facial artery and veins proximal to and distal to the submandibular gland, the posterior belly of digastric, stylohyoid, and styloglossus muscles). The mobility afforded by the mandibular osteotomy allows for delicate dissection of the underlying structures, identifying first the nerve to the mylohyoid and the lingual nerve, then the submandibular ganglion and duct (Figure 6).

**Figure 7:**
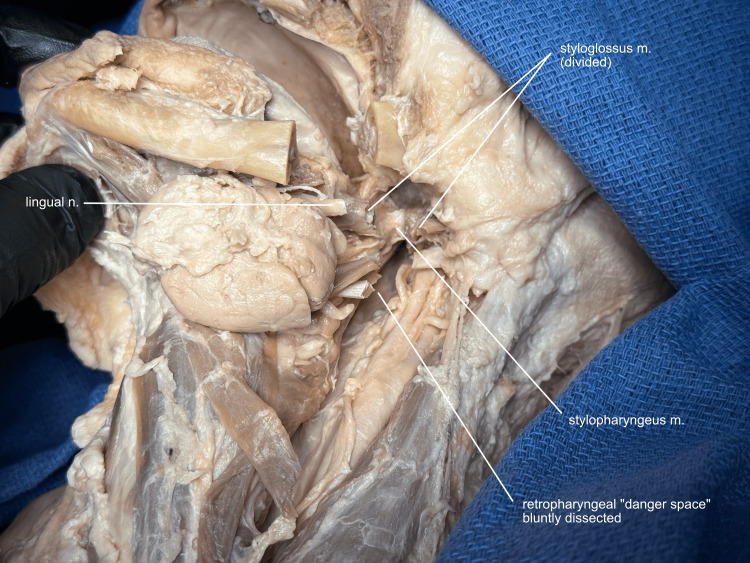
Lateral and posterior pharyngotomy To optimize educational opportunities, the lingual nerve is divided proximal to the mandibular osteotomy to preserve the spatial relationships of the lingual nerve with the submandibular gland, duct, and ganglion. The only lateral structure preventing mandibular rotation is now the pharyngeal wall. The stylopharyngeus muscle is visible medial to the styloglossus. The palatopharyngeus, medial to this, is not visible in this view. The retropharyngeal space is bluntly dissected in preparation for the division of the posterior pharyngeal wall. m, muscle; n, nerve

After these anatomical relationships are appreciated, the lingual nerve is divided about one centimeter proximal to the submandibular ganglion, along with the nerve to the mylohyoid. The only structure now remaining deep into the osteotomy is the lateral pharyngeal wall, including the stylopharyngeus, palatopharyngeus, and pharyngeal constrictor muscles (Figure 7).

**Figure 8:**
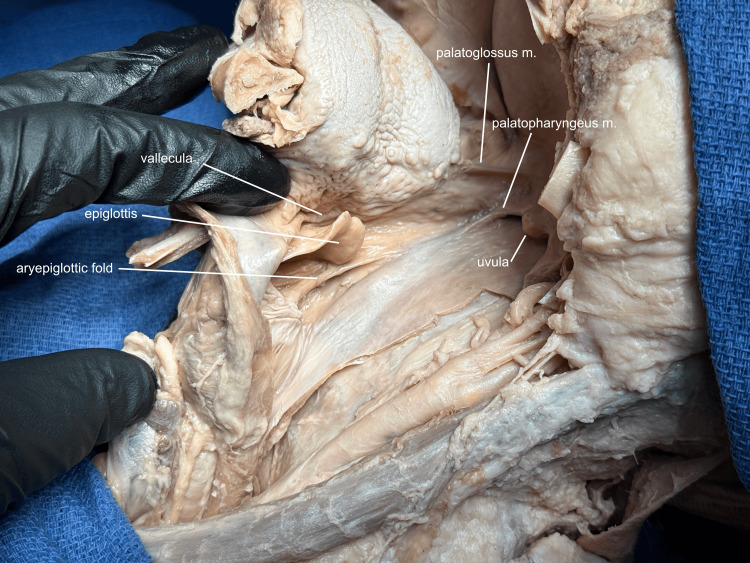
Mandibular rotation The oral pharyngeal incision is now carried down into the esophagus, and the anterior mandible is rotated 90 degrees to allow full visualization of the oropharynx and laryngopharynx. m, muscle

Returning to the danger space, the posterior cricoid cartilage and the greater horns of the hyoid bone are palpated to determine the midline of the posterior pharyngeal wall, and the oral incision is continued down this line to the esophagus. The anterior mandible can now be rotated 90 degrees to allow full visualization of the base of the tongue, the vallecula, piriform recess, glottis, and epiglottis, and their relationships to the laryngopharynx and oropharynx (Figure 8).

## Discussion

**Figure 9:**
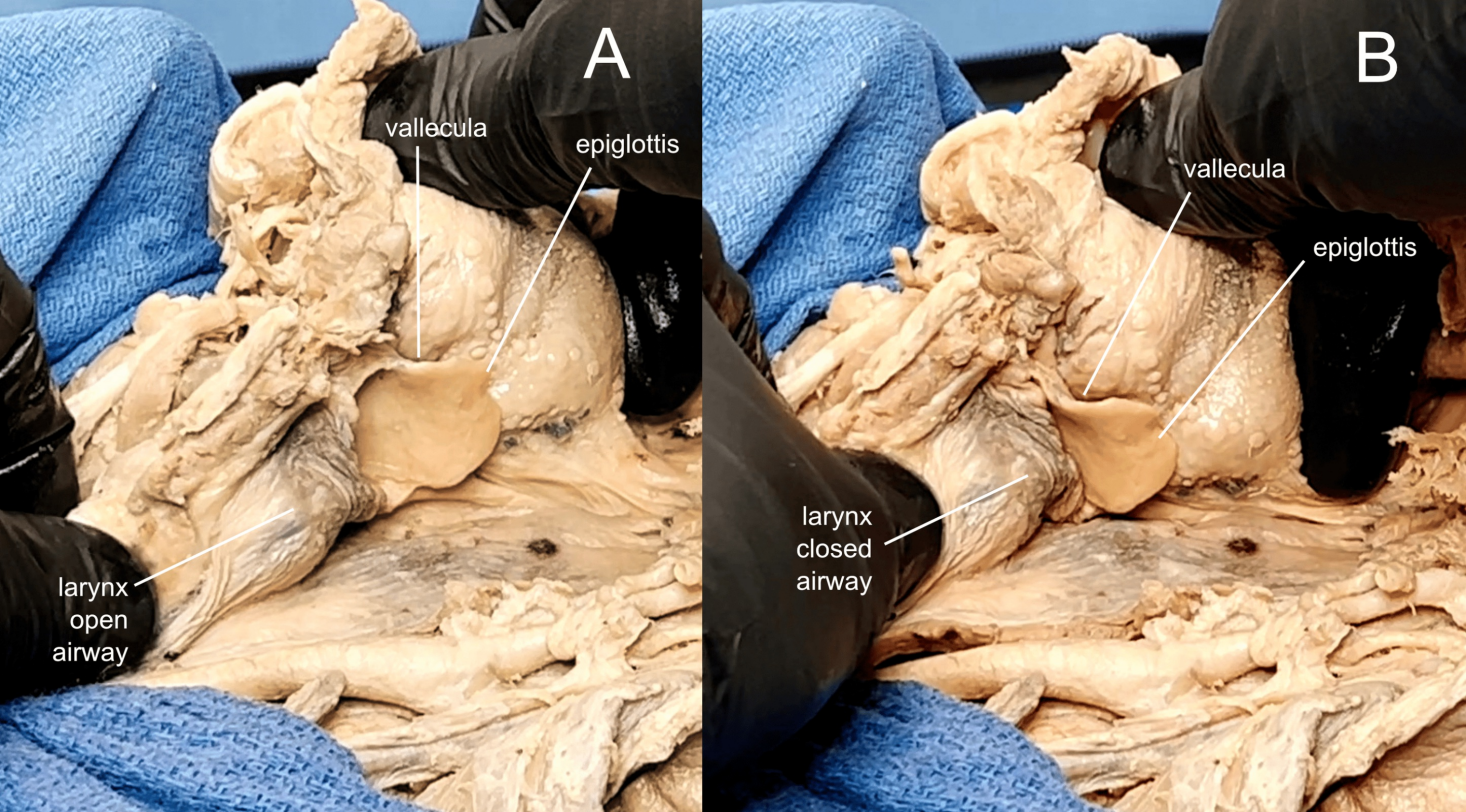
Mechanics of airway protection The ease of access to the tongue and larynx allows for the visualization of airway protection. An open airway is seen in frame (A), and extrinsic glottic closure is simulated in frame (B) by moving the tongue posteriorly and the larynx anteriorly and superiorly.

Despite there being a broad base of literature on airway protection and swallowing, there is a gap in the integration of gross anatomy with function. In a review of dysphagia in neurological disease, while there is much detail regarding the neurological pathways and associated neurological conditions, there is little mention of specific anatomical structures as they relate to function and dysfunction [6]. The physiological phases of swallowing are well described in the literature. Clinical reasoning steps have been laid out to diagnose which specific phase is the cause of the dysphagia, but a demonstration of the mechanical changes, such as seen with an in situ anatomical dissection, is lacking. For example, in a recent research paper on swallowing disorders, the function of the tongue is described solely as preparing and transporting the bolus of food or liquid [7]. While certainly true of much of the tongue's function, it overlooks a key functional relationship between the tongue and epiglottis regarding airway protection. This relationship is very evident using the functional larynx dissection we describe. Other detailed descriptions of the phases of swallowing are correlated with general regions, such as the oral cavity and pharynx, but not so much with individual muscles. In a description of the events evident on a barium swallow, an overview of swallowing notes that the "epiglottis tilts back" [8]. On a fluoroscopic view, this may be all one can conclude, but with the anatomical view afforded by our functional larynx dissection, it is not only evident what is occurring, but also how. How the vertical fibers of the inferior pharyngeal constrictor, stylopharyngeus, and palatopharyngeus muscles would elevate the larynx can be visually appreciated. The dynamic relationship between the tongue and epiglottis can be seen by moving them both, viewing the vallecula and epiglottis from lateral, superior, and posterior views. The function of the horizontally oriented styloglossus muscle, although divided to rotate the mandible, can be seen to pull the base of the tongue posteriorly onto the epiglottis. Combining this action with the upward pull of suprahyoid muscles and the vertical pharyngeal muscles, the anatomic basis of extrinsic glottic closure is easily appreciated (Figure 9). 

**Figure 10:**
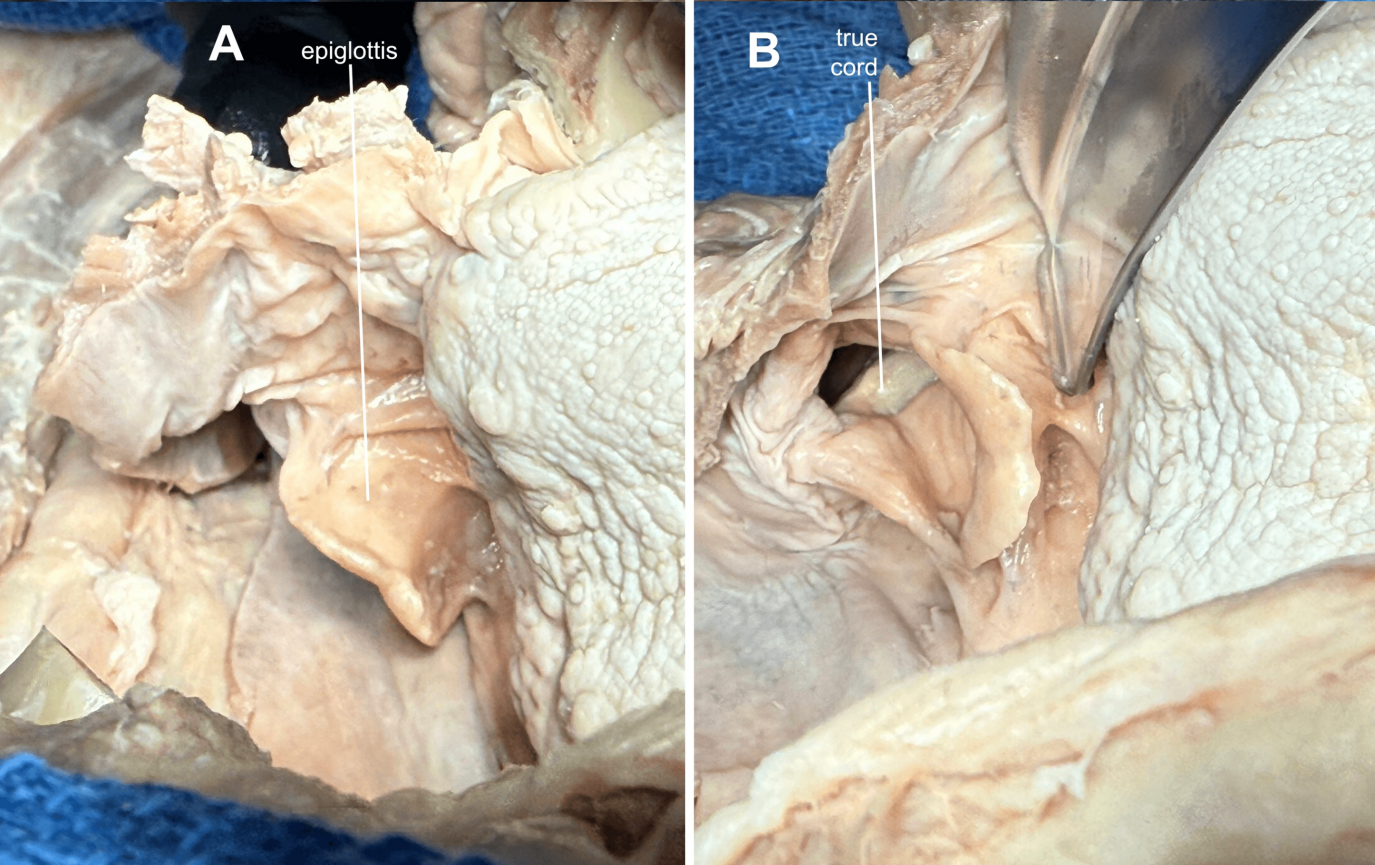
Anatomy related to intubation Viewing the tongue and vallecula from multiple angles, students can appreciate how a laryngoscope blade can draw the epiglottis anteriorly to expose the cords.

A sagittal hemisection of the head, usually performed on at least one cadaver during gross anatomy courses, adds a unique view of the geniohyoid muscle. The significance of this muscle can often be lost on students, but when considered in light of the functional larynx dissection, its role can be understood: to pull the hyoid and larynx anteriorly, while the styloglossus muscles pull the tongue posteriorly, folding down the elastic epiglottis. Rotating and viewing the larynx from multiple angles allows students to reproduce the view they would see during intubation. Unlike the experience in a simulation lab, this approach allows for multiple angles of view and hands-on palpation of real anatomy. Using a laryngoscope blade, students can appreciate how the anterior elevation of the blade opens the glottis safely and effectively (Figure 10).

**Figure 11:**
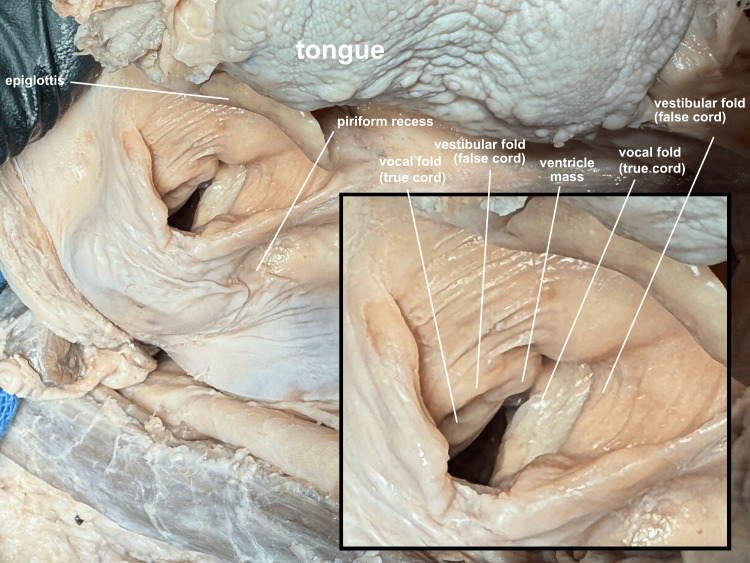
Intrinsic laryngeal anatomy The mandibular rotation exposure of the larynx allows for manipulation of the larynx to optimize views of intrinsic structures. A nodule is noted in this donor's left anterior ventricle (the space between the true and false cords).

The intrinsic anatomy of the larynx can also be better appreciated by manipulating the larynx to optimize views. Viewing the laryngeal ventricle in an intact larynx can be difficult. This area, between the true and false cords, has significant clinical correlations, such as laryngoceles, infection, and carcinoma [9]. Being difficult to appreciate in a disarticulation and appearing only as a groove in a hemisected larynx, it is not discussed much in most anatomy textbooks. With the rotational exposure of the larynx, it can be seen as a significant space in which cysts or masses could develop, affecting speech or airway function. Of note, the donor in this study's dissection has a mass in her left anterior ventricle (Figure 11).

**Figure 12:**
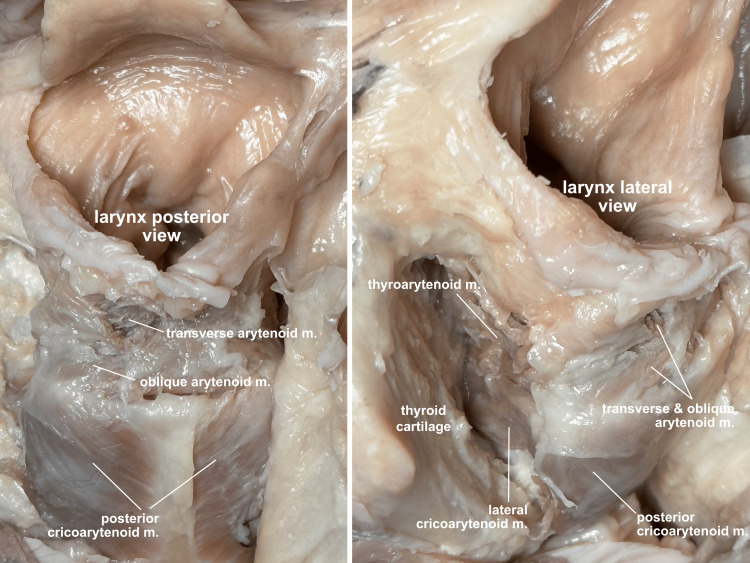
Intrinsic laryngeal muscles The functional larynx dissection allows for an in situ exposure of both posterior and lateral intrinsic laryngeal muscles. Visualizing the opposing fibers of the posterior and lateral cricoarytenoid muscles, students can appreciate how the posterior rotates the arytenoid cartilages to open (abduct) the cords, while the lateral rotates them the opposite way to close (adduct) the cords. The thyroarytenoid muscle is seen running from the anterior thyroid cartilage to the arytenoid cartilage. m, muscle

Another aspect of the larynx that students often struggle with is the intrinsic muscles. The ability to rotate the larynx with this approach provides both posterior and lateral access to the intrinsic muscles. The mucosa overlying the posterior aspect of the cricoid cartilage and the piriform recess is reflected, and the space between the thyroid cartilage and the intrinsic muscles is gently dissected. In addition to the arytenoid and posterior cricoarytenoid muscles, which are usually seen with the disarticulation approach, the lateral cricoarytenoid muscle can also be seen with this approach. Students can visualize the opposing actions of the posterior and lateral cricoarytenoid muscles on the arytenoid cartilages and cords (Figure 12).

**Figure 13:**
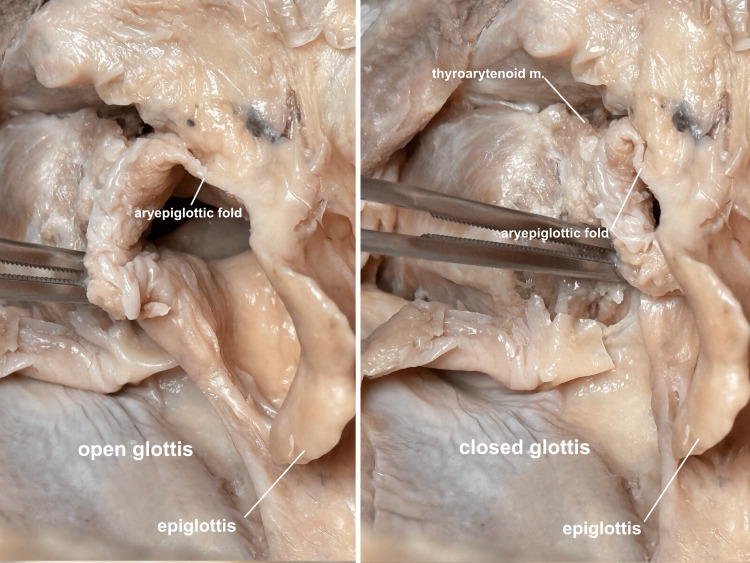
Intrinsic glottic closure Intrinsic glottic closure can be simulated, and the participating muscles visualized: the transverse and oblique arytenoid muscles drawing the posterior arytenoid cartilages together, the lateral cricoarytenoid muscles rotating the cords closed, and the thyroarytenoid muscles drawing the arytenoid cartilages toward the anterior thyroid cartilage, bunching up the aryepiglottic folds. m, muscle

The thyroarytenoid muscle can also be seen from the lateral viewpoint, and intrinsic glottic closure can be simulated to help students understand how this muscle, along with the lateral cricoarytenoid and arytenoid muscles, closes the glottis prior to the folding down of the epiglottis (Figure 13).

**Figure 14:**
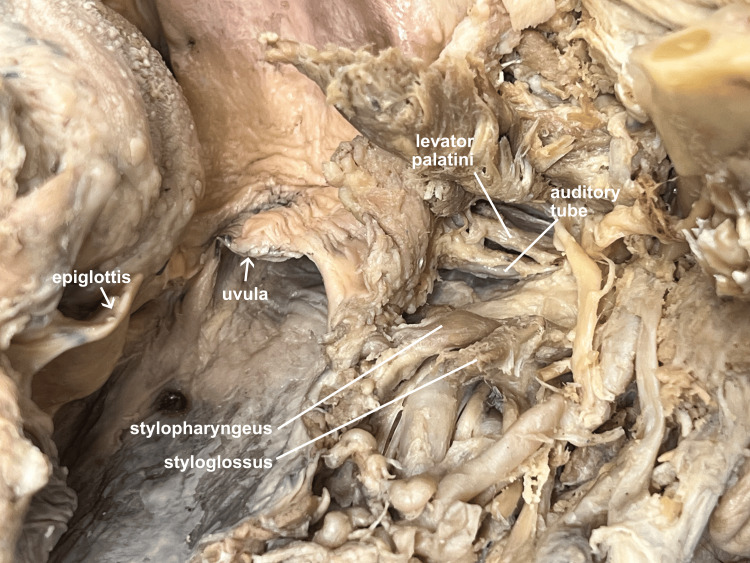
Ventral anterior view of auditory tube The lateral mandibular rotation approach provides access to visualize the levator palatini and auditory tube, from a ventral anterior approach.

Other structures that can be appreciated with this approach include the auditory (Eustachian) tube and the levator palatini muscle. With retraction of the angle of the mandible, gentle elevation of the medial pterygoid muscle, and digital palpation of the torus tubarius, posterior and superior to the soft palate, the auditory tube and levator palatini can be identified (Figure 14).

**Figure 15:**
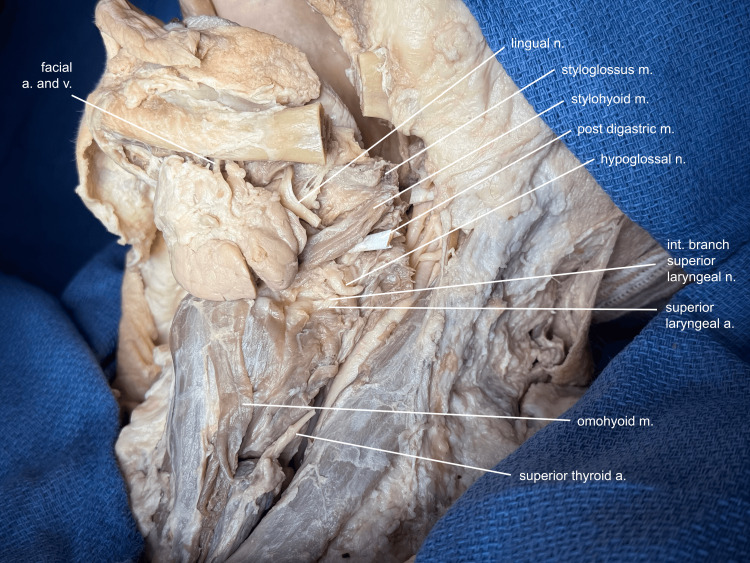
Returning to native position Rotating back to the original position, the divided structures can again be viewed and identified. a, artery; v, vein; m, muscle; n, nerve

The relationship of the larynx to adjacent head and neck structures can be appreciated as the mandible is rotated back to its native position, and the divided structures are again viewed in their normal position (Figure 15).

The mandibular rotation affords a functional, visually impactful representation of both intrinsic and extrinsic laryngeal anatomy, in one dissection approach.

Nine second-year students, training to be teaching assistants in the upcoming year's anatomy course, assisted with the performance of the mandibular rotation technique for a functional larynx dissection. In a survey with a five-point ordinal scale from strongly disagreeing to strongly agreeing, all nine students either agreed or strongly agreed that the dissection approach improved their understanding of laryngeal anatomy, the mechanics of airway protection and swallowing, and the structures visualized during intubation.

## Conclusions

Traditional methods of teaching laryngeal anatomy fail to maintain the structural relationships pertaining to laryngeal function. As such, students often have trouble understanding the function of muscles, nerves, and other structures involved with airway protection and swallowing. The pharyngotomy and mandibular rotation technique we describe addresses this shortcoming by exposing the intact larynx from a lateral approach, keeping its relationships to the laryngopharynx, oropharynx, tongue, and soft palate intact.
